# A randomized prospective study: assessment of transient ureteral stenting by mono-J insertion after primary URS and stone extraction (FaST 3)

**DOI:** 10.1007/s00240-021-01277-z

**Published:** 2021-06-19

**Authors:** Alina Reicherz, Hannah Westhues, Lorine Häuser, Patricia Wenzel, Joachim Noldus, Peter Bach

**Affiliations:** Department of Urology, Marien Hospital, Ruhr-University Bochum, Hölkeskampring 40, 44625 Herne, Germany

**Keywords:** Ureteroscopy, Urolithiasis, Flexible ureteroscopy, Double-J, Transient ureteral stenting using a mono-J ureteral catheter

## Abstract

To compare the outcome of a short-term insertion of a mono-J catheter for 6 h following ureteroscopic stone removal to a conventional double-J catheter. This single-center academic study (Fast Track Stent study 3) evaluated stenting in 108 patients with urinary calculi after ureterorenoscopy. Patients were prospectively randomized into two study arms before primary ureterorenoscopy: (1) mono-J insertion for 6 h after ureterorenoscopy and (2) double-J insertion for 3–5 days after ureterorenoscopy. Study endpoints were stent-related symptoms assessed by an ureteral stent symptom questionnaire (USSQ) and reintervention rates. Stone sizes and location, age, operation duration, BMI, and gender were recorded. Of 67 patients undergoing ureterorenoscopy, 36 patients were analyzed in the double-J arm and 31 patients in the mono-J arm. Mean operation time was 27.5 ± 1.3 min versus 24.0 ± 1.3 min, and stone size was 5.2 mm versus 4.5 mm for mono-J versus double-J, respectively (*p* = 0.06 and *p* = 0.15). FaST 3 was terminated early due to a high reintervention rate of 35.5% for the mono-J group and 16.7% for the double-J group (*p* = 0.27). One day after ureterorenoscopy, USSQ scores were similar between the study arms (Urinary Index: *p* = 0.09; Pain Index: *p* = 0.67). However, after 3–5 weeks, the Pain Index was significantly lower in those patients who had a double-J inserted after ureterorenoscopy (*p* = 0.04). Short-term insertion of mono-J post-ureterorenoscopy results in similar micturition symptoms and pain one day after ureterorenoscopy compared to double-J insertion. The reintervention rate was non-significant between the treatment groups most likely due to the early termination of the study (*p* = 0.27). Ethics approval/Trail Registration: No. 18-6435, 2018

**Supplementary Information:**

The online version contains supplementary material available at 10.1007/s00240-021-01277-z.

## Introduction

The necessity for stenting after ureterorenoscopy (URS) has been widely discussed, and practice differs internationally [1]. Potential benefits and adverse effects of stent insertion have to be balanced. Stents are often inserted prophylactically, as ureteral manipulation can cause swelling and obstruction. Meta-analyses found no difference in stone-free rates (SFR) [2–4]. However, patients who were stented after URS were more likely to complain of irritative micturition symptoms compared to those who did not receive a stent.

European and American guidelines state that urologists can omit stenting after uncomplicated URS and complete stone removal [5, 6].

The Fast Track Stent Studies (FaST 1–3) are successive, prospective randomized trials with similar designs to evaluate drainage after URS [7–10].

**Fig. 1:**
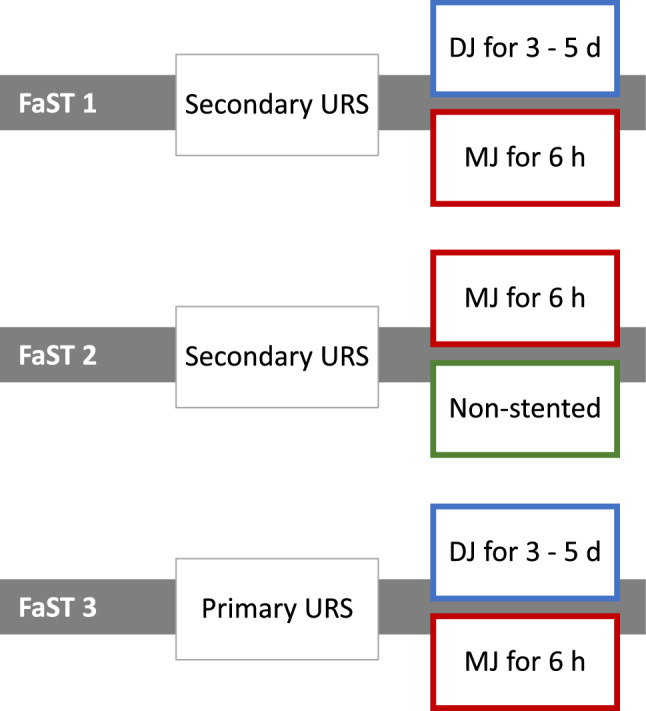
Overview FaST (1, 2 and 3) trials. Patients in all trials were enrolled and randomized before URS. Each study comprised of two arms comparing drainage after stone extraction. In the FaST 1 and 2 trials, patients were pre-stented. Patients in the FaST 1 and 2 studies completed the USSQ one day before and 3–5 weeks after URS. FaST 3 study collected USSQ results one day and 3–5 weeks after URS

FaST 1 compared a double-J (DJ) insertion for 3–5 days to a mono-J (MJ) insertion for 6 h after secondary URS (Fig. 1). Patients with a MJ reported fewer irritative micturition symptoms and pain and reported a better performance in everyday life, while the reintervention rate was higher compared to patients who had a DJ inserted [7]. FaST 2 compared a MJ placement with an indwelling time of 6 h to a tubeless procedure after secondary URS (Fig. 1). Patients from both study arms experienced a significantly improved QoL after URS. However, reintervention rates were significantly lower in patients with a MJ (1.6 %versus 13.3%) [8].

Furthermore, the necessity of stenting before URS is a matter of debate. The American guidelines provide a strong recommendation against routine stent placement [5]. Moreover, The European and German Urological Associations state that routine stenting before URS is not necessary. However, they note that pre-stenting improves outcomes and decreases complications (Level 1b) [6, 11].

Current studies show that German practices often deviate from recommendations in the guidelines [12]. For example, data from the German BUSTER study shows that pre-stenting is performed in 70% of patients before stone extraction in Germany [13].

The present work aims to analyze the safety of MJ insertion after primary URS and whether stent-associated symptoms can be reduced by inserting a MJ instead of a DJ after URS.

## Methods

The FaST 3 recruited patients from 07/2018 to 09/2020. Ethics approval was obtained through the Ruhr University of Bochum (No. 18-6435).

In this academic, single-center trial, patients were assigned via block randomization with an allocation ratio of 1:1 to a DJ placement for 3–5 days or a short-term MJ insertion for 6 h. Patients at least 18 years of age who had a URS planned for ureteral or renal stones smaller than 15 mm were eligible for this study. Those with a single kidney or concurrent urinary tract infections were not eligible. Additionally, if patients were ineligible for primary URS due to strictures, the SFR was below 80% after primary URS, operation time exceeded one hour, or complications occurred (American Association for the Surgery of Trauma-Organ Injury Scale (AAST) Grade 2), patients were secondarily excluded. Termination criterion was a reintervention rate of > 30%. Single-dose parenteral antibiotic therapy was administered before surgery. Six surgeons, not blinded to randomization, performed URS using a 6.4 Fr/4.2 Fr channel semirigid instrument by Olympus^®^ and a 9.9 Fr flexible URS instrument by Olympus^®^. For flexible URS, access sheaths (12 Fr, Coloplast^©^) were used. A holmium laser (LISALaser^®^) was used for lithotripsy and set to 80 Hz and 0.3 Joule to dust stones. After stone removal, SFR was checked fluoroscopically, and stents were inserted. Depending on the study arm, either a DJ by Coloplast^®^ (6 Fr, 26 cm) or a MJ by Coloplast^®^ (VORTEK^®^, 6 Fr) was placed. Nurses pulled out the MJs on the ward after 6 h. After URS, patients received diclofenac (50 mg orally, twice daily) and tamsulosin off label (0.4 mg orally, once daily) over three days.

Primary endpoints were urinary symptoms on day 1 and 3–5 weeks after URS assessed using the USSQ. Secondary endpoints were reintervention rates and pain, QoL, work performance, sexual concerns and additional problems, also assessed by the USSQ [10]. The USSQ is a validated questionnaire with good internal consistency and test–retest reliability. It comprises 6 sections with 38 questions regarding stent-associated symptoms. A sample size of 53 patients in each group is required to demonstrate a difference of 15% in the Urinary Symptoms Index between populations with a statistical power of 80% [10]. Intention-to-treat analysis was applied in the present study. Crossovers were transferred from the original to the new treatment group. Missing data were deleted pairwise. We used GraphPad Prism 5 for statistical analysis. Patients' characteristics were examined using Student's *t* test and Fisher's exact test. USSQ results and reintervention rates were compared using a Mann–Whitney *U* test, Student's *t* test and Fisher's exact test. The level of significance was defined as *p* < 0.05.

## Results

**Fig. 2:**
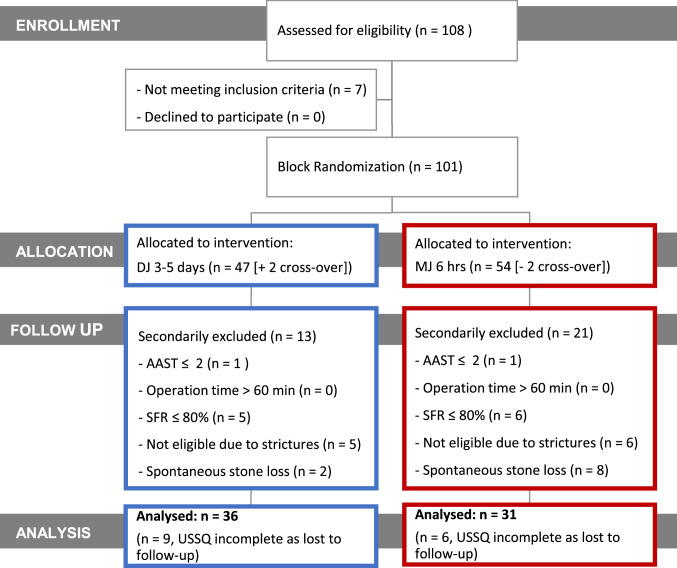
FaST 3 study flowchart

One hundred eight patients were assessed for eligibility. Seven patients had to be excluded because they did not meet the inclusion criteria (*n* = 7). None of the patients declined to participate (*n* = 0). One hundred one patients met the inclusion criteria and gave informed consent to participate (Fig. 2). Patients were randomly allocated to two interventions after URS: DJ drainage for 3–5 days (*n* = 47) or MJ drainage for 6 h (*n* = 54). Two patients who were initially randomized into the MJ arm were crossed over into the DJ group by the surgeon. Thirty-four patients were secondarily excluded. Thirty-six patients were analyzed in the DJ arm and thirty-one in the MJ arm. In fifteen patients, USSQ results were incomplete because they were lost to follow-up. Patient characteristics are shown in Table 1. The MJ and DJ groups were not statistically different in terms of stone size, stone density, number of semirigid and flexible URS procedures, age, operation duration, or body mass index (BMI) (Table 1).

**Table 1:** Patient characteristic

			95% CI	*p* value
	MJ	DJ	MJ vs. DJ	MJ vs. DJ
Gender, (%)				0.79
Male	71.0	66.6		
Female	29.0	33.3		
Age [years], mean ± SD	47 ± 2.5	49 ± 2.6		0.57
BMI [kg/m^2^], mean ± SD	28.6 ± 0.9	28.3 ± 0.7	− 2.1 to 2.7	0.81
Stone size [mm], mean ± SD [range]	5.2 ± 0.4 [0–8]	4.5 ± 0.3 [2–12]	− 1.6 to 0.26	0.15
Stone density [HU], mean ± SD	672 ± 70	680 ± 53	− 183.5 to 167.2	0.93
Stone location﻿, (%)				
Distally	96.8	86.1	–	
Lumbar	0	2.8	–	
Proximally	3.2	11.1	–	
No stone found	0	0	–	
Operation time [min], mean ± SD [range]	27.5 ± 1.3 [5–60]	24.0 ± 1.3 [5–50]	− 7.15 to 0.19	0.06
Flexible URS﻿, (%)﻿	9.7	0	–	0.09

### Reinterventions

Due to high reintervention rates in the MJ group, early termination of the study was necessary. Reintervention rates did not differ significantly according to the randomization group (Table 2). Reasons for reintervention were symptomatic hydronephrosis (visual analogue scale > 7) or fever (> 38.5 °C) following URS. Reinterventions were required in 35.5% (11 out of 31) of patients in the MJ group and 16.7% (6 out of 36) in the DJ population (*p* = 0.27). In case of reintervention, a DJ stent by Coloplast^©^ (7 Fr, 26 cm) was placed. Follow-up procedures were carried out within 48 h of stone removal.

**Table 2:** USSQ results one day after and 3-5 weeks after stone removal and reintervention rates

Results: USSQ indices and GQ† for randomized groups one day after URS				
	Mean ± SD (lower and upper 95% CI)		*p* value	
	MJ	DJ	MJ vs. DJ	
Urinary Index	26.6 ± 6.6 (24.0–29.1)	29.3 ± 8.5 (26.1–32.5)	0.14	
Pain Index	20.0 ± 12.1 (15.4–24.6)	22.3 ± 13.8 (16.9–27.7)	0.67	
General Health Index	Not inquired one day after URS		–	
Work Index	Not inquired one day after URS		–	
Sexual Index	Not inquired one day after URS		–	
GQ	3.3 ± 1.8 (2.4–4.1)	3.9 ± 1.7 (3.1–4.7)	0.22	

Results: USSQ indices and GQ† for randomized groups 3–5 weeks after URS				
	Mean ± SD (lower and upper 95% CI)		*p* value	
	MJ	DJ	MJ vs. DJ	
Urinary Index	16.9 ± 4.3 (15.1–18.6)	16.3 ± 5.3 (14. 3–18.4)	0.43	
Pain Index	14.4 ± 9.9 (10.4–18.4)	10.9 ± 7.5 (7.9–13.9)	**0.04**	
General Health Index	7.8 ± 2.2 (6.9–8.8)	8.7 ± 2.8 (7.6–9.8)	0.13	
Work Index	3.5 ± 1.4 (2.9–4.1)	3.7 ± 1.5 (2.7–4.6)	0.90	
Sexual Index	2.6 ± 1.4 (1.9–3.4)	2.6 ± 1.2 (1.9–3.2)	0.94	
GQ	2.7 ± 2.0 (1.9–3.4)	3.4 ± 1.6 (2.7–4.0)	0.06	

Results: reintervention rate				
	Rate [%]		95% CI	*p* value
	MJ	DJ	MJ vs. DJ	MJ vs. DJ
	35.5	16.7	−0.02 to 0.40	0.27

†Question GQ: “in the future, if you were advised to have another stent inserted, how would you feel about it?”

Subgroup analysis of mono-J patients (Table 3) did not identify pre- or intraoperative parameters that increased postoperative reintervention risk.

**Table 3:** Comparison of patients who required versus patients who did not require reintervention after URS and short-term MJ insertion

	MJ group no reintervention (*n* = 11)	MJ group reintervention (*n* = 20)	*p* value
Age, mean ± SD	49 ± 3.2	42 ± 3.5	0.19
Sex, (%)			0.43
Male	65	82	
Female	35	18	
BMI, mean ± SD	29 ± 0.9	26 ± 0.7	0.06
Stone size, mean ± SD	4.7 ± 0.4	6.2 ± 0.9	0.07
Stone localization (distal), (%)	100	90	0.35
Surgery time, mean ± SD	27 ± 1.6	29 ± 2.0	0.50
Flexible URS device, (%)	5	18	0.28
Intraoperative macrohematuria, (%)	0	22	0.09

### USSQ: urinary concerns

**Fig. 3:**
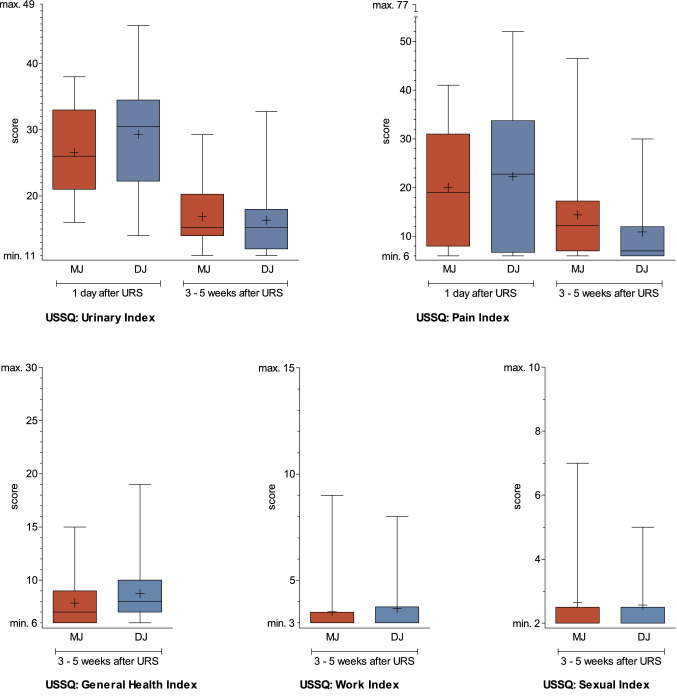
FaST 3 Study USSQ indices (Urinary, Pain, General Health, Work and Sexual Index). Urinary Index: (1) DJ: *n* = 24, MJ: *n* = 28, *p* = 0.14; (2) DJ: *n* 27, MJ: *n* = 25, *p* = 0.43. Pain Index: (1) DJ: *n* = 28, MJ: *n* = 29, *p* = 0.67; (2) DJ: *n* 27, MJ: *n* = 26, *p* = 0.04. General Health Index: DJ: *n* = 27, MJ: *n* = 25, *p* = 0.13. Work Index: DJ: *n* = 12, MJ: *n* = 21, *p* = 0.90. Sexual Index: DJ: *n* = 14, MJ: *n* = 17, *p* = 0.94. Boxplot: whiskers: min and max, mean shown as  + , median shown as a horizontal line

On the day after stone removal, the Urinary Index did not differ between the MJ and DJ groups (*p* = 0.04; Fig. 3, Table 2). All patients reported that they had “a little bit" to "moderate" of micturition complaints after intervention (Q U10: *p* = 0.76). Patients from both study arms responded that they would have "mixed feelings" to question U11: “If you were to spend the rest of your life with the urinary symptoms, if any, associated with the kidney problem just the way they are, how would you feel about it?" (Q U11: *p* = 0.21). After 3–5 weeks, patients from both study arms answered the same question and stated they would feel "pleased" to "delighted" (Q U11: *p* = 0.43). Moreover, micturition complaints decreased in both study arms without a significant difference between the groups.

### USSQ: pain concerns

Pain was reported by 62.1% of DJ versus 67.9% of MJ patients one day after URS (*p* = 0.78). Three to five weeks after URS, the incidence of pain was 61.5% for patients of the MJ group and reduced to 44.4% for those in the DJ group (*p* = 0.27).

On day one after URS and stone removal, the Pain Index did not differ significantly between the study arms (*p* = 0.67; Table 2, Fig. 3). After 3–5 weeks, patients from the DJ group showed a lower Pain Index (*p* = 0.04).

### USSQ: general health concerns

The drainage applied after URS had no impact on the General Health Index 3– 5 weeks after URS (*p* = 0.13; Table 2 and Fig. 3).

Question GQ asked about patients' feelings if they were advised to have a stent inserted in future. On the day after URS, all patients stated they would have "mixed feelings" to feel "mostly satisfied" (Q GQ: *p* = 0.22; Table 2). During the follow-up questioning, MJ and DJ patients reported they were "mostly satisfied" (Q GQ: *p* = 0.06) (Table 2).

### USSQ: return to work

Functional limitations were not an issue in either of the treatment arms. Immobilization was rare (MJ:1.1 versus DJ: 1.2 days; *p* = 0.51). Patients experienced a limitation in daily activities for MJ: 2.8 versus DJ: 2.6 days (*p* = 0.74). Regardless of drainage, the Work Index was similar (*p* = 0.90; Table 2 and Fig. 3).

### USSQ: additional concerns

After URS, most patients did not report symptoms associated with urinary tract infection. This finding was related to neither MJ nor DJ insertion (Q A1: *p* = 0.98). The type of urinary drainage did not result in additional visits to a doctor (Q A3: *p* = 0.37) or repeat hospital admissions following therapy (Q A4: *p* = 0.82).

### Ineligibility for primary URS

10.3% of patients randomized in the FaST 3 were not eligible for primary URS due to ureteral strictures and thus were secondarily excluded. The assessment of eligibility for URS was at the urologist's discretion.

## Discussion

To our knowledge, the FaST 3 is the first prospective randomized study comparing a MJ insertion to a DJ insertion after primary URS. This study is preceded by the FaST 1 and 2, which evaluated short-term MJ placement, DJ placement, and omission of stenting after secondary URS [7, 8]. We demonstrated that patients benefit from significantly improved urinary symptoms, pain, general health and workability if a MJ is transiently placed or no stent is inserted. However, reintervention rates differed significantly, with no reintervention in the DJ arm, 5.0% in the MJ arm and 13.3% if stenting is omitted.

### Postoperative stenting

American, European and German guidelines agree that routine stenting after URS is not necessary [5, 6, 11, 12]. A Cochrane analysis including 23 trials and 2,656 patients addressing the effect of postoperative ureteral stent placement after uncomplicated URS showed that stenting slightly reduces the risk of unplanned return visits and has little to no impact on secondary interventions and postoperative pain. However, they outlined that the certainty of the evidence is low to very low [14].

The European guidelines advise that "a ureteral catheter with a shorter indwelling time (one day) may also be used", referring to an editorial comment by Moon et al. [6, 15].

FaST 3 compared a transient MJ to a DJ placement after primary URS. QoL in terms of micturition symptoms and pain was comparable one day after URS across both randomized groups. 3–5 weeks after URS, the Pain Index was significantly higher in the MJ group, while micturition symptoms did not differ significantly. No difference was found regarding general health, sexual complaints, or the ability to work. The reintervention rate was higher when a MJ was transiently inserted (32.2% versus 19.4; *p* = 0.27), though without significance. A prospective observational, international multicentre study by the Endourologic Society (CROES) showed that stenting after removal of both ureteral and renal stones reduces the risk of complications (*p* < 0.001) [1]. In a retrospective study, Merlo et al. compared a DJ placement (2–4 weeks) to a MJ placement (up to 24 h) to an omission of stenting after URS. One month after URS, patients with transient MJ placement reported LUTS incidence, hematuria, pain, fever and required hospital care significantly less often than patients who had received a DJ.

Literature suggests that contrary to the guidelines, DJ stent use after URS is very common. An analysis of postoperative DJ stenting by the CROS study group showed that the USA, China, Canada and Japan placed DJ stents postoperatively in more than 90% of patients [1]. A survey among German urological departments showed that after primary URS, 79.6% of urologists inserted a DJ, 7.3% inserted a MJ, and 3.6% omitted a stent. After secondary URS, urologists inserted a DJ in 62.2% of cases, a MJ in 10.5% and did not insert a stent in 14.0% [16].

We conclude that in our study cohort, routine placement of a MJ for 6 h after URS is not safe considering the high reintervention rate.

### Primary URS

Guidelines claim that stenting before uncomplicated URS is not necessary [5, 11]. "However, pre-stenting facilitates ureteroscopic management of stones, improves the SFR, and reduces intra-operative complications" [6]. The CROES URS Global Study (*n* = 8189 patients with ureteral calculi) showed that in 12.7% of cases before semirigid URS and 37.8% before flexible URS, a DJ was inserted preoperatively [17]. The share of pre-stented patients was exceptionally high in Germany. In the FaST 3 protocol, 10.3% of all patients required pre-stenting. Those patients were excluded as they were ineligible for primary URS. Comparing the reintervention rates of the FaST 3 to those of FaST 1 and 2 allow for a comparison of reintervention rates between primary and secondary URS in the same clinical setting [7, 8]. If a DJ was inserted, no reinterventions were necessary for pre-stented patients, while the reintervention rate was 16.7% after primary URS (*p* = 0.002). If a MJ was inserted, reinterventions were necessary for 5.0% of pre-stented patients, while the reintervention rate was 35.5% after primary URS (*p* < 0.0001). Comparing the existing literature is difficult: while most studies report about complications versus the need for reintervention, a database study on postoperative stenting, including a heterogeneous population of 11,885 patients, reported that 16.0% needed retreatment, including readmission [18]. The operative reintervention rate in the FaST 3 collective is high; an explanation might be the study's prospective character. Urologists were required to stick to the study protocol; however, the eligibility for a primary URS was at the surgeons' discretion.

Remarkably, treatment modalities (high rate of pre-stenting) deviate in a highly developed country with a well-equipped health system like Germany, and this could be due to financial incentives and a missing endourological department system [16].

Our study has limitations. First, surgeons might have been biased as they were aware of the treatment allocation. A second limitation is that patients were aware of this allocation, which possibly affected USSQ results. FaST 3 was preliminarily terminated due to the high reintervention rate in both groups, thus did not achieve the required sample size.

Given the high rate for postoperative reintervention, short-term mono-J drainage after primary URS is only reasonable for a selected patient collective and depends on surgeons' assessment.

## Supplementary Information

Below is the link to the electronic supplementary material.

Supplementary file1 (PDF 101 KB)

## Abbreviations

AAST: American Association for the Surgery of Trauma (ureter)
BMI: Body mass index
CROES: Clinical Research Office of Endourological Society
DJ: Double-J stent
MJ: Mono-J stent
FaST: Fast track stent studies
QoL: Quality of life
SFR: Stone free rate
URS: Ureterorenoscopy
USSQ: Ureteral stent symptom questionnaire
